# Simvastatin prevents alveolar bone loss in an experimental rat model of periodontitis after ovariectomy

**DOI:** 10.1186/s12967-014-0284-0

**Published:** 2014-10-01

**Authors:** Xin-chen Xu, Hui Chen, Xi Zhang, Zan-jing Zhai, Xu-qiang Liu, An Qin, Er-yi Lu

**Affiliations:** Department of Prosthodontics, Shanghai Ninth People’s Hospital, Shanghai Jiao Tong University School of Medicine, Shanghai Key Laboratory of Orthopaedic Implant, Shanghai Key Laboratory of Stomatology, 639 Zhizaoju Road, Shanghai, 200011 China; Department of Orthopaedic, Shanghai Ninth People’s Hospital, Shanghai Jiao Tong University School of Medicine, Shanghai Key Laboratory of Orthopaedic Implant, 639 Zhizaoju Road, Shanghai, 200011 China

**Keywords:** Simvastatin, Alveolar bone loss, Periodontitis, Osteoporosis, Maxillary

## Abstract

**Background:**

Periodontitis is an inflammatory disease characterized by the loss of connective tissue and alveolar bone. There is an increasing evidence that periodontitis is associated with a number of chronic disease, including osteoporosis. Periodontitis and osteoporosis are both bone destructive diseases and of high prevalence in adult population. Osteoporosis could increase some inflammatory factors that also participate in the progression of periodontitis, so as to facilitate the alveolar bone resorption. Simvastatin, specific inhibitor of 3-hydroxy-3-methylglutaryl-coenzyme reductase, is of pleiotropic effects including anti-catabolic and anabolic effect on bone metabolism. This study aimed to explore the local and systemic effect of simvastatin on maxillary in rats with both osteoporosis and periodontitis.

**Methods:**

Thirty-six 4-month-old female Sprague Dawley rats were randomly assigned to six groups: sham group, ligature group, ovariectomized (OVX) + ligature group, local simvastatin administration to OVX + ligature rats (local simvastatin group), oral simvastatin administration to OVX + ligature rats (oral simvastatin group), local and oral simvastatin administration to OVX + ligature rats (L&O simvastatin group). One month after OVX, ligatures were placed on the maxillary first (M1) and second molars (M2) for 4 weeks on all rats except those in the sham group, followed by simvastatin treatment for 2 months. The maxillae, serum, and femurs were collected for further examination including micro-computed (micro-CT) tomography, hematoxylin and eosin (H&E) staining, tartrate-resistant acid phosphatase (TRAP) staining, enzyme-linked immunosorbent assays (ELISA), and the three-point bending test.

**Results:**

Local simvastatin administration increased alveolar crest height and prevented local alveolar bone loss without alteration of systemic bone loss. Oral administration prevented local and systemic bone loss with no effect on alveolar crest height.

**Conclusions:**

Our results indicate that simvastatin has the potential of promoting bone formation and reducing alveolar bone loss in maxillary following ovariectomy (OVX) and ligature placement in rats.

## Background

Periodontitis is a destructive disease that targets tooth-supporting structures through complex and multifactorial pathogenic processes [1]. The resorption of the alveolar crest bone initiated by periodontitis can gradually lead to tooth loss and edentulousness, which in turn results in continued alveolar bone loss (ABL) and resorption of the remaining residual ridge. There is increasing evidence that periodontitis is associated with a number of chronic diseases such as osteoporosis [1,2]. Osteoporosis is a skeletal disorder characterized by compromised bone strength, predisposing the bone to an increased risk of fracture [3]. Dramatic advances in the understanding of bone metabolism has brought us a new prospective that possible pathways by which systemic bone loss, such as osteoporosis, may contribute to more rapid alveolar bone loss in periodontitis [2,4]. A possible mechanism could relate to a more susceptible environment for bacterial deposit in oral cavity caused by the hormonal changes associated with postmenopausal osteoporosis. Since osteoporosis could increase some inflammation factors, and some of these factors are also participated in the progression of periodontitis [5–7]. Therefore, the administration of compounds that can prevent bone loss and inhibit inflammatory cytokines has the potential to treat both periodontitis and osteoporosis.

Statins are specific inhibitors of 3-hydroxy-3-methylglutaryl-coenzyme reductase that are traditionally used to inhibit the production of cholesterol in cardiovascular diseases [8–12]. However, statins have pleiotropic therapeutic effects including vasodilatory, antithrombotic, antioxidant, anti-inflammatory, and immunosuppressive actions [13–15]. In 1999, Mundy G et al. [16] first reported that statins were potent stimulators of bone formation *in vitro.* In 2011, Moon HJ et al. [17] proved that simvastatin acted as an osteoclastogenesis inhibitor by suppressing reactive oxygen species-mediated signaling pathways. Therefore, simvastatin has both anti-catabolic and anabolic effect on bone metabolism. Indeed, Vaziri H et al. [18] have evaluated the effect of simvastatin on ligature-induced bone resorption in the mandible of ovariectomized rats. Their results demonstrated the protective effects of local simvastatin administration on a periodontal attachment apparatus and ABL. Based on their research, we aimed to explore the effect of simvastatin on maxillary and compare the effects of local and oral simvastatin administration in a rat model with both periodontitis and osteoporosis.

## Methods

### Animals and study design

All animal procedures were performed under guidelines approved by the Ethics Committee and the Animal Care and Use Committee of Shanghai Jiao Tong University School of Medicine (HKDL[2013]33). Thirty-six 4-month-old female Sprague–Dawley rats (approximate weight 230 to 250 g) from the laboratory animal science company (Slaccas, Shanghai, China) were selected for this study. All rats, housed in filter-top cages in a temperature and humidity controlled room (23 ± 1°C and 60 ± 5%, respectively) with a 12-h light–dark cycle, were allowed a 1-week acclimation period before surgery.

The rats were divided according to body weights, so that each study group had similar mean body weight values. To explore the effect of simvastatin on alveolar bone, the rats were divided into 6 groups of 6 rats each (Table 1): sham group, ligature group, ovariectomized (OVX) + ligature group, local simvastatin administration to OVX + ligature rats (local simvastatin group), oral simvastatin administration to OVX + ligature rats (oral simvastatin group), local and oral simvastatin administration to OVX + ligature rats (L&O simvastatin group).

**Table 1:** **The experimental design and related treatment was as follows**

**Group no.**	**Surgery**	**Treatment**	**Dose and duration**
1	sham	vehicle	saline and two months
2	ligature	vehicle	saline and two months
3	OVX + ligature	vehicle	saline and two months
4	OVX + ligature	local simvastatin	0.8 mg/0.05 mL and two months
5	OVX + ligature	oral simvastatin	25 mg/kg and two months
6	OVX + ligature	local and oral simvastatin	0.8 mg/0.05 mL, 25 mg/kg and two months

Under anesthesia consisting of 10% chloral hydrate (4 mL/kg, intraperitoneal injection), bilateral ovariectomies were performed in groups 3 to 6, while sham surgeries were performed in sham group and ligature group. Four weeks after ovariectomy or sham surgery, silk sutures (3/0) (Johnson & Johnson Medical [China] Ltd., Shanghai, China) were placed bilaterally into the gingival sulci of the first and second molars (M1 and M2, respectively) in groups 2 to 6 under anesthesia to experimentally induce periodontitis for 4 weeks. During the ligature period, the rats were checked once a week to ensure success of the experimentally induced periodontitis. Seto H et al. [19] reported that the bone loss caused by experimental periodontitis could persist for 45–90 days after removal of the ligatures. Before starting simvastatin treatment, the ligatures were removed at the end of the experimentally induced periodontitis phase. The doses of simvastatin for local and oral administration were chosen within the range found to be safe in rats, and the administration lasted for 2 months [20,21]. Saline or simvastatin was injected into the periodontal pocket of M1 and M2 bilaterally in all groups except the oral simvastatin group, while the oral simvastatin and L&O simvastatin groups received an oral administration of 25 mg/kg simvastatin every day (Table 1).

### Micro-computed tomography (CT) scanning and assessment of the alveolar bone

Both sides of the maxillae were fixed in 4% paraformaldehyde for 48 hours and scanned using a cone beam micro-CT system (Skyscan1176, Skyscan, Kontich, Belgium) at Soochow University Orthopaedic Institute. The x-ray generator was set at a voltage of 50 KV with a current of 500 μA and a fixed exposure time of 900 ms, which produced images with a voxel size of 9 × 9 × 9 μm^3^. Scans were reconstructed to generate three-dimensional models and microstructure indicators of bone volume/tissue volume (BV/TV), trabecular thickness (Tb.Th), trabecular separation (Tb.Sp) and trabecular number (Tb.N) were determined using a three-dimensional region of interest (ROI) under a calculated Hounsfield Unit grayscale threshold value by Skyscan analysis software (Version: 1.10, CT Analyser, Skyscan, Belgium). The ROI was a cuboidal bone body that encompassed the roots. The ROI length extended from the most mesial aspect of M1 root to the most distal aspect of M2 root, the width extended from the most buccal aspect to the most palatal aspect of M1 or M2 root, and the height extended from the most apical aspect of M1 or M2 root to the most coronal part of alveolar bone crest (ABC).

With the help of the Mimics software (Materialise NV, Belgium), ABL, represented by the linear distance of the cemento-enamel junction (CEJ) to the ABC, indicated the severity of periodontitis during the clinical examinations, and was measured in millimeters. Briefly, ABL was measured at four points on each tooth (M1 and M2), which were the mesiolingual (ML), mesiobuccal (MB), distolingual (DL), and distobuccal (DB) regions. Additionally, a high ABL value meant heavy bone loss, whereas a low ABL value indicated a healthier alveolar bone.

### Bone histology observation

After micro-CT analysis, the maxillae were decalcified with 10% Ethylene Diamine Tetraacetic Acid disodium salt for about 2 months, embedded in paraffin, and cut into 5-μm-thick serial sagittal sections. Half of these sections were stained with hematoxylin and eosin (H&E) for descriptive analysis, and the rest were stained with tartrate-resistant acid phosphatase (TRAP, Sigma, St. Louis, MO, USA) to identify osteoclasts on the bone surface. All sections were observed and photographed under a Nikon microscope (Nikon eclipse 90i, Japan) and analyzed using the ImageJ 1.46r software (National Institutes of Health, MD, USA).

### Bone specific serum biomarkers

Whole blood samples were collected from the heart on the day of sacrifice. Immediately, all samples were centrifuged to obtain the serum and were stored at −80°C until analysis [22]. Serum osteocalcin (OCN) was measured using a RatMAGNETIC Bone Pane Kit (RBNMAG-31 K, Millipore, St Charles, MO, USA). Additionally, serum tartrate-resistant acid phosphatase 5b (TRAP5b) was measured using rat ELISA Kit (SB-TR102, IDS, Fountain Hills, AZ, USA).

### Biomechanical testing

The rats’ right femurs were collected and stored at −20°C wrapped with phosphate buffered saline soaked gauze. Before starting the three-point bending test, the femurs were transferred to an ambient temperature to thaw. The femurs were analyzed in a materials testing machine (InstronE10000, Instron Corporation, Norwood, MA, USA), using a three-point bending procedure. Femurs were compressed until failure at a displacement rate of 3 mm/min (span = 20 mm) with the anterior side down, and all force and displacement data were recorded until the specimen was broken. Then, caliper measurement of the periosteal and endosteal diameters was performed on the broken femurs, and bending stress (MPa), bending strain (mm/mm), and Young’s modulus (MPa) were calculated, assuming the cross-sectional geometry of the femurs were ellipses.

### Statistical analysis

All values are presented as the mean ± standard deviation (SD). One-way analysis of variance with Bonferroni test was performed for measuring statistically significant differences between groups for all outcomes. The Statistical Package for the Social Sciences version 17.0 software package (SPSS Inc., Chicago, IL, USA) was used for all analyses. A value of p < 0.05 or p < 0.01 was considered significant.

## Results

### Micro-CT analysis of alveolar bone microarchitecture and measurement of ABL

**Figure 1:**
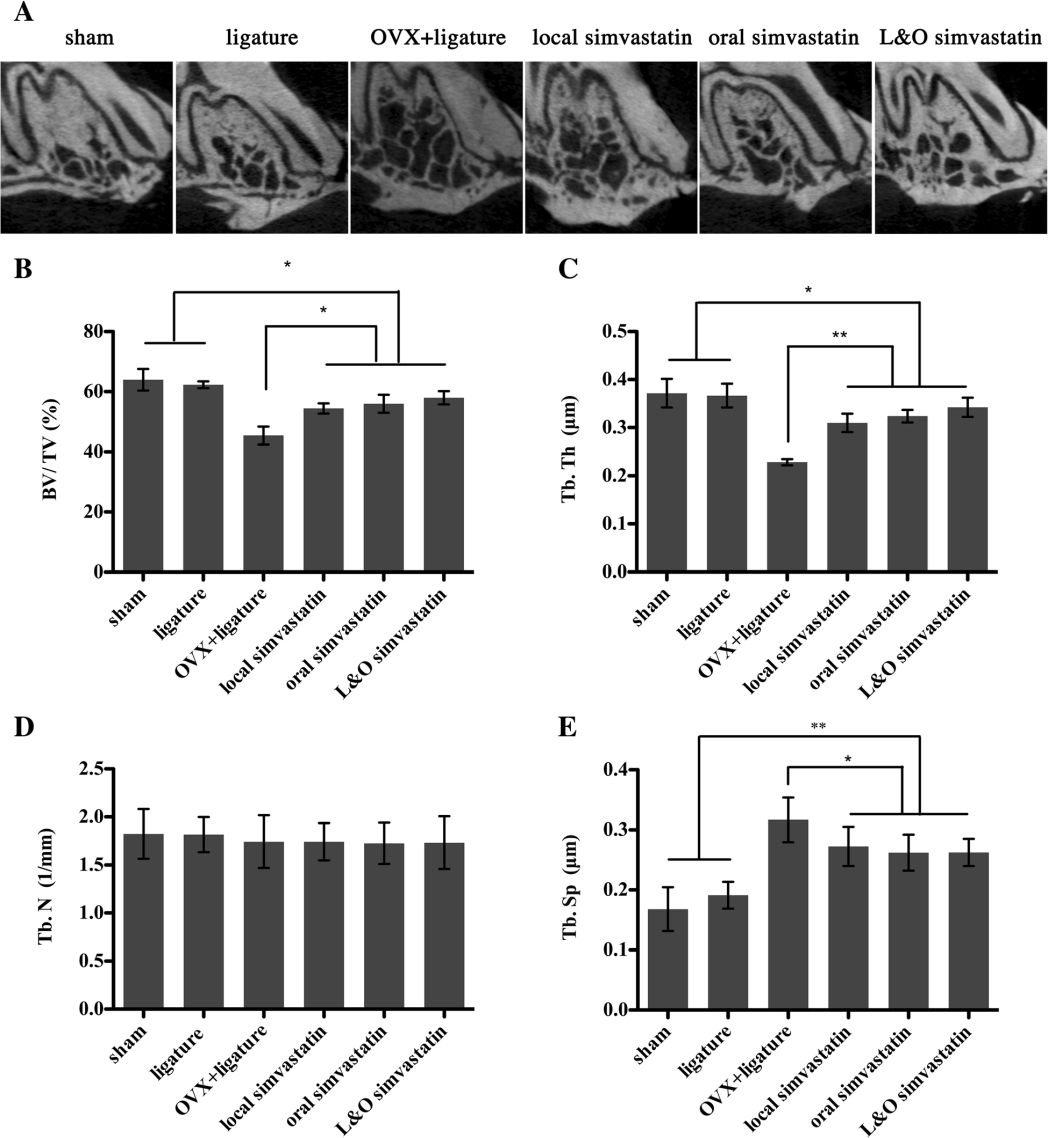
**Effect of simvastatin on maxilla by micro-CT analysis of bone volumetric parameters. (A)** Longitudinal-sectional micro-CT images of the first molar (M1) in the maxilla. **(B–E)** Analysis of micro-CT volumetric parameters: bone volume/tissue volume (BV/TV), trabecular thickness (Tb.Th), trabecular separation (Tb.Sp), and trabecular number (Th.N). Values are expressed as the mean ± the standard deviation (N = 12; * indicates p < 0.05, ** indicates p < 0.01).

Figure 1A shows the typical longitudinal-sectional CT images of the M1 in maxilla. It is obvious that the trabecular and cortical thicknesses are greater in the sham and ligature groups than in the OVX rats. The local simvastatin, oral simvastatin, and L&O simvastatin groups were associated with increased BV/TV, increased Tb.Th, and decreased Tb.Sp compared to the OVX + ligature group (Figure 1B, C, and E). There was no significant difference in Tb.N (Figure 1D) among the groups.

**Figure 2:**
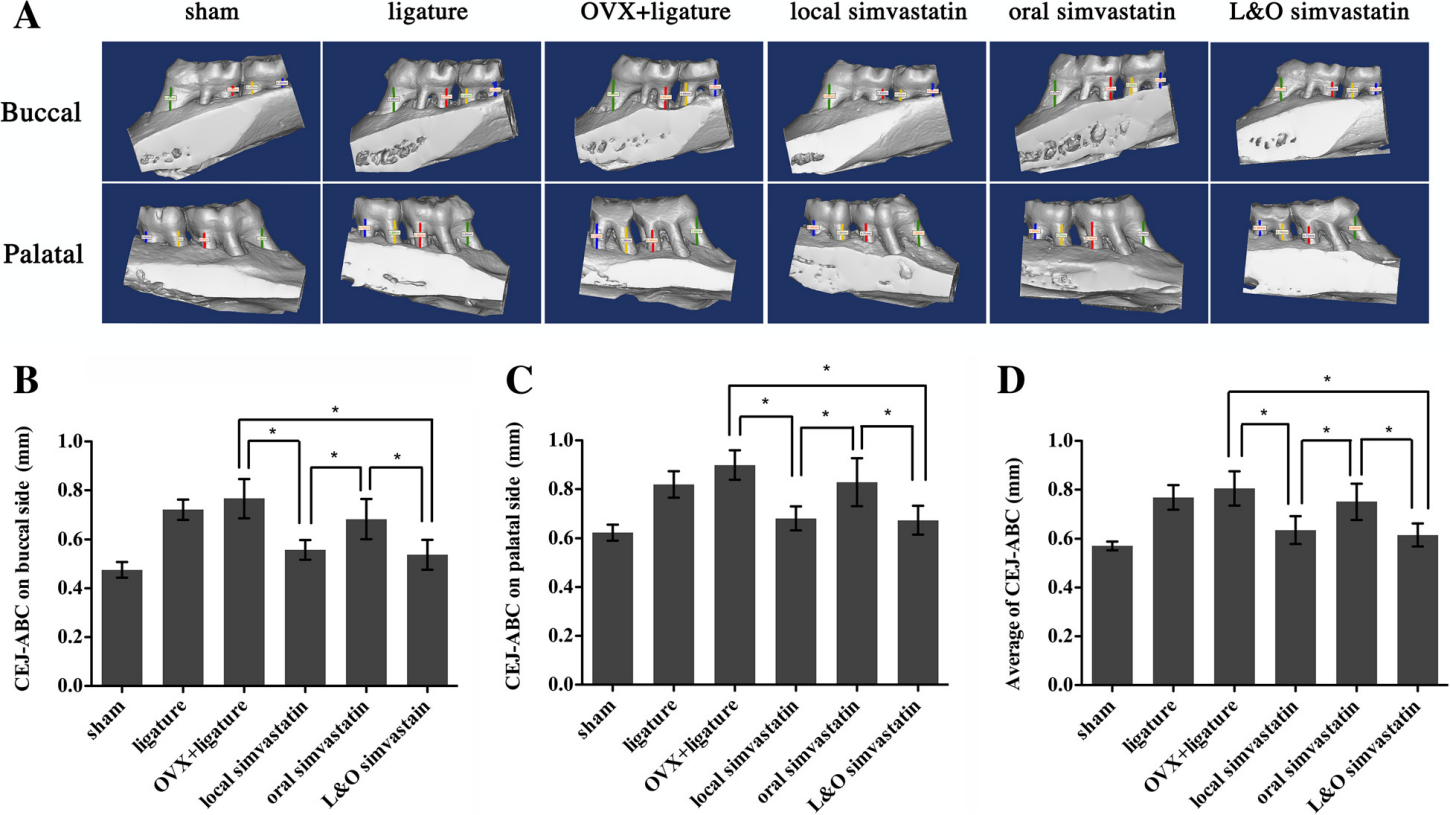
**Effect of simvastatin on maxilla of cemento-enamel junction (CEJ) to alveolar bone crest (ABC). (A)** Buccal and palatal sides of the maxilla, where alveolar bone loss (ABL, colored lines) was measured from the CEJ to the ABC at four points: mesiolingual (ML), mesiobuccal (MB), distolingual (DL), and distobuccal (DB) regions for first maxillary molar (M1) and second maxillary molar (M2). The region of interest (ROI) is a cuboidal bone body that encompasses the roots of the M1 and M2. **(B–D)** Analysis of the CEJ-ABC linear distance after 2 months of simvastatin or placebo treatment. Values are expressed as the mean ± the standard deviation (N = 12; * indicates p < 0.05).

An obvious decrease in alveolar crest height could be observed in the rats with ligature placement, especially on the palatal side (Figure 2A). Notably, the local simvastatin and L&O simvastatin groups had partially prevented the reduction in alveolar crest height in the buccal side (27%/28%, local/L&O), the palatal side (21%/23%, local/L&O), and the buccal and palatal sides (23%/24%, local/L&O; p < 0.05; Figure 2B, C, and D) compared to the OVX + ligature group. Compared to the oral simvastatin group, the local simvastatin and L&O simvastatin groups recovered the reduction in alveolar crest height in the buccal side, the palatal side, and the buccal and palatal sides by 23%/25%, 16%/17%, and 18%/19% (local/L&O), respectively (p < 0.05; Figure 2B, C, and D). Taken together, local or oral simvastatin administration prevented alveolar trabecular bone loss, while local simvastatin administration partially increased the alveolar crest height.

### Histomorphometric analysis of the alveolar bone

**Figure 3:**
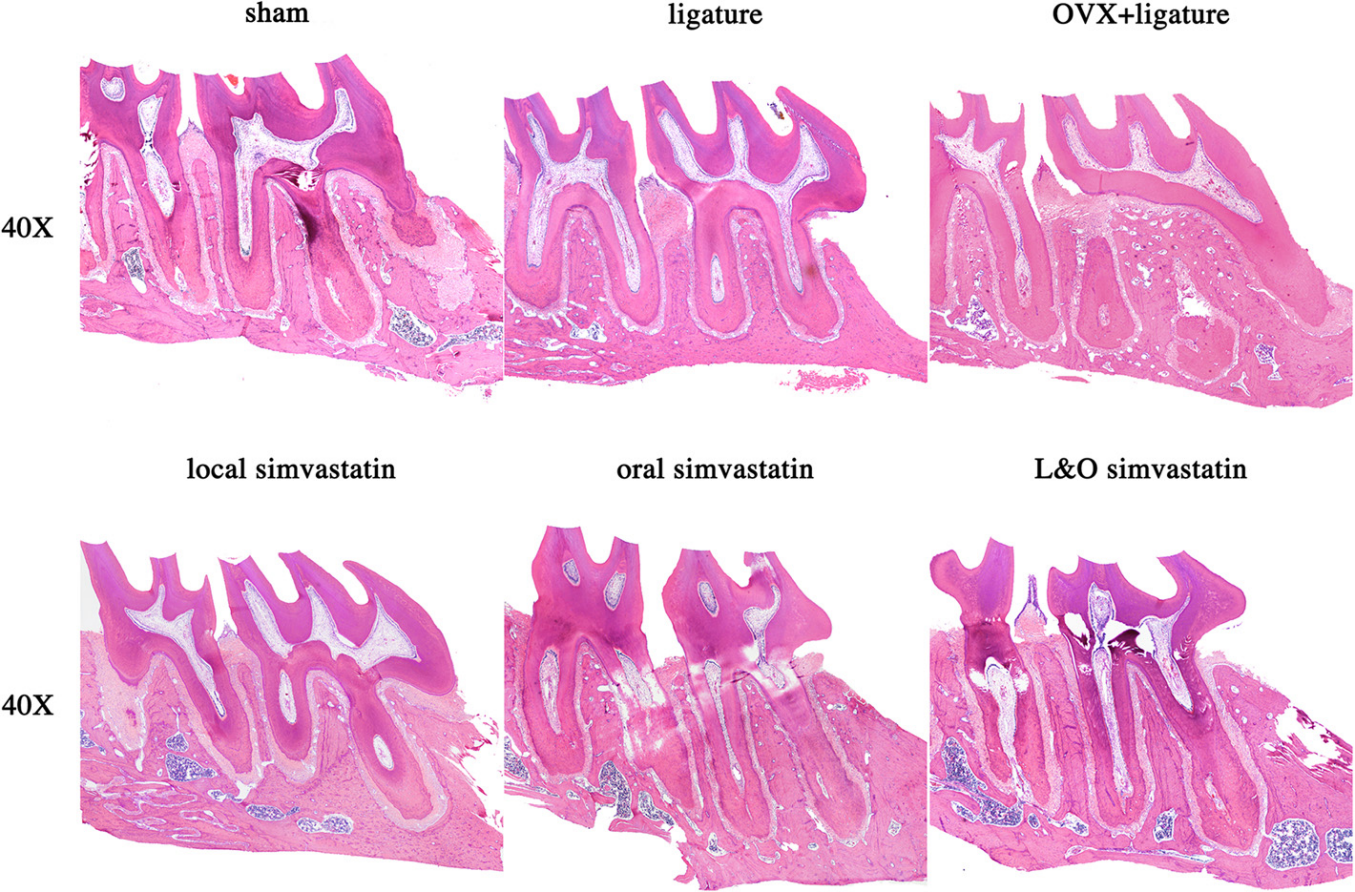
**Descriptive analysis of H&E staining in paraffin sections.** The images show sagittal sections of each group at 40× magnification.

After analyzing the H&E staining sections, it was obvious that the unattached periodontal ligament between M1 and M2 could be found in the groups having ligature placement (Figure 3). Consistent with the micro-CT results, the amount of bone tissue was higher in all of the simvastatin administration groups. In addition, the alveolar crest height was relatively higher in the local and L&O simvastatin groups, indicating a potential protective effect of local simvastatin administration on preserving alveolar crest height (Figure 3).

**Figure 4:**
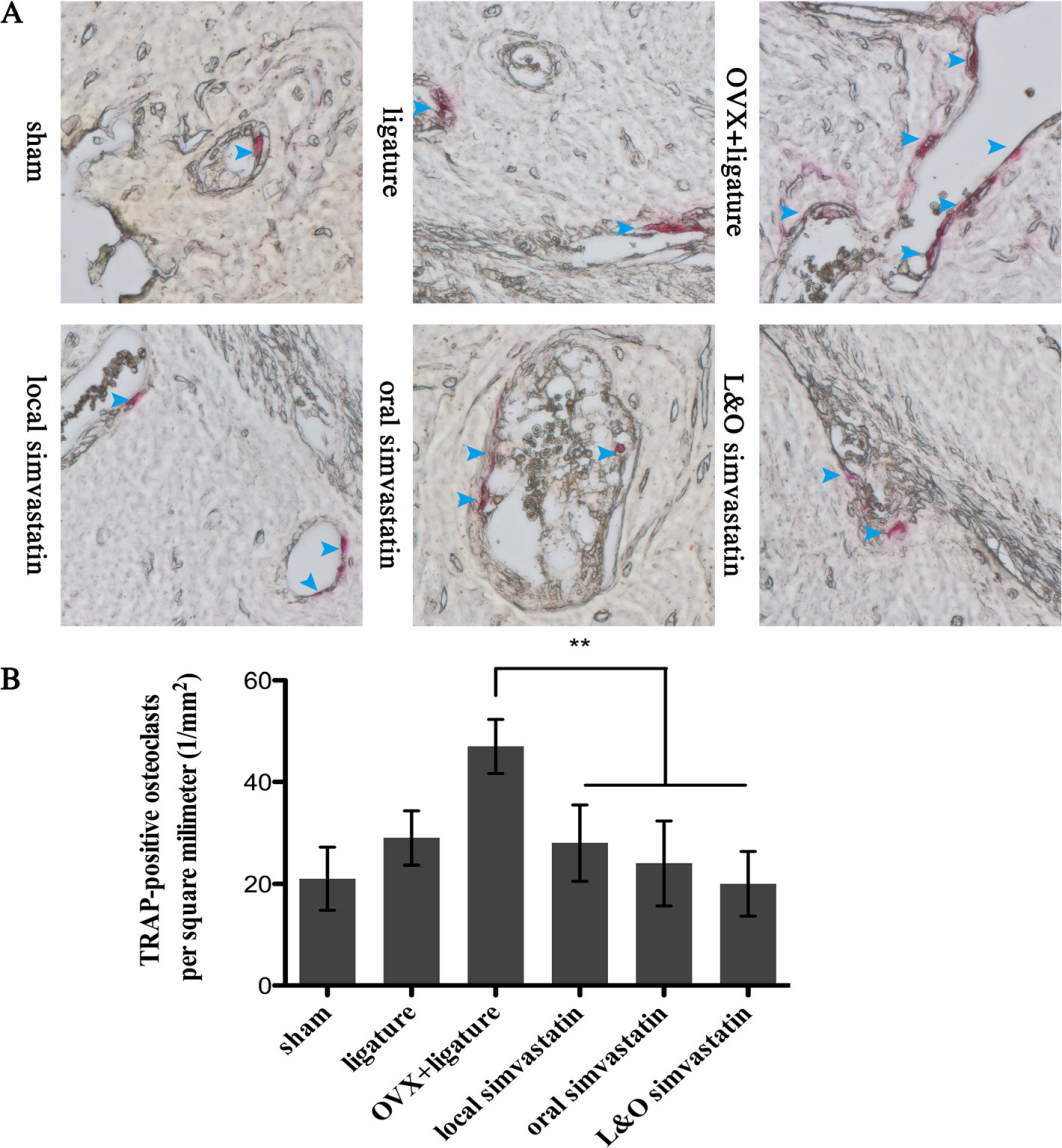
**Analysis of the number of osteoclasts in a 1 × 1 square millimeter. (A)** 400 × magnified images of alveolar bone after TRAP staining and the multinuclear osteoclasts are stained red (blue arrows). **(B)** Analysis of the number of osteoclasts in a 1 mm^2^ area. Values are expressed as the mean ± the standard deviation (N = 36; ** indicates p < 0.01).

Six sections of every sample were selected for analysis of the number of TRAP-positive osteoclasts. The number of osteoclasts was obviously decreased by approximately 49%, 52%, and 58% in the local simvastatin, oral simvastatin, and L&O simvastatin groups compared to the OVX + ligature group, suggesting that simvastatin could inhibit osteoclast formation (p < 0.01; Figure 4A, B).

### Serum biochemical analysis of OCN and TRAP5b

**Figure 5:**
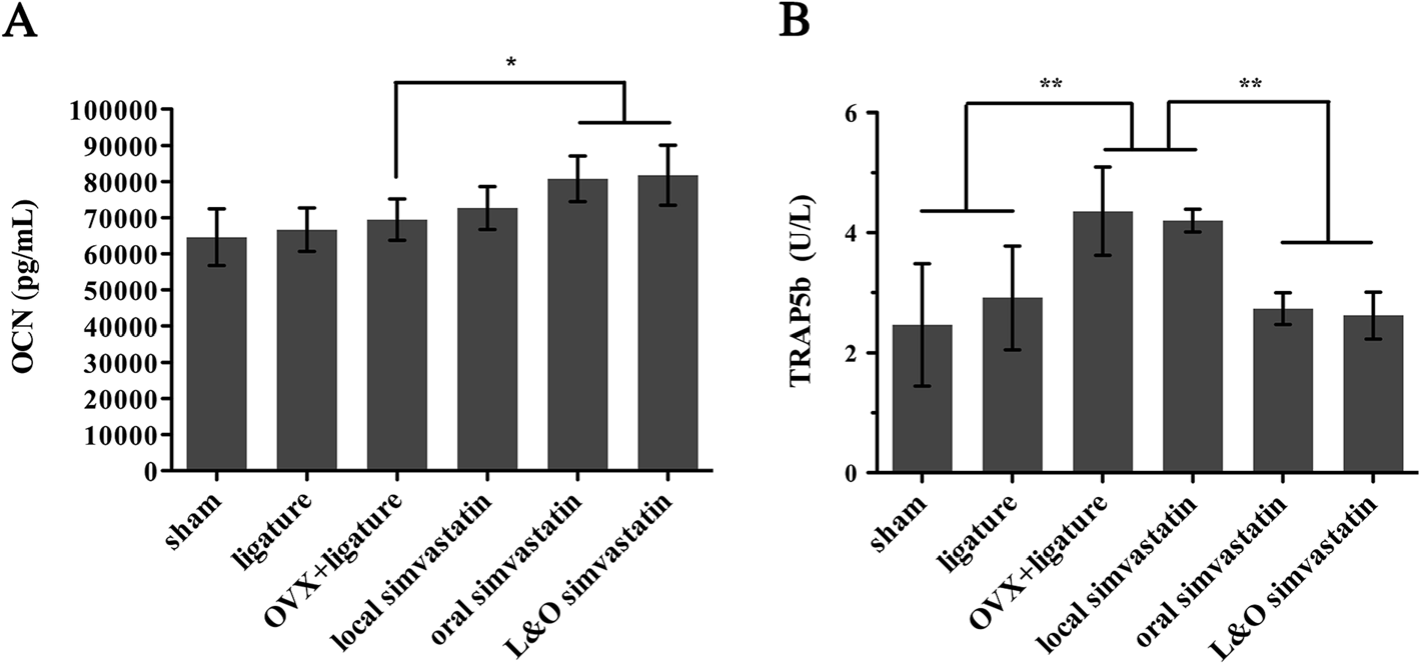
**Analysis of bone formation and resorption serum biochemical markers after simvastatin treatment. (A)** Changes in the serum levels of the bone formation marker osteocalcin (OCN) are shown after 2 months of simvastatin or saline treatment. **(B)** Changes in the serum levels of the bone resorption marker tartrate-resistant acid phosphatase 5b (TRAP5b) are shown after 2 months of simvastatin or saline treatment. Values are expressed as the mean ± the standard deviation (N = 6; * indicates p < 0.05, ** indicates p < 0.01).

The therapeutic effect of simvastatin on preventing bone loss was further examined by analyzing serum bone-remodeling biomarkers, including the osteoblast marker OCN and the osteoclast marker TRAP5b. As shown in Figure 5A, oral simvastatin and L&O simvastatin groups increased the serum OCN level by about 12% compared to the level in the OVX + ligature group (p < 0.05). However, local simvastatin group did not change the serum OCN level remarkably. Additionally, it was obvious that oral simvastatin and L&O simvastatin groups reduced the serum TRAP5b level by about 46% compared to the level in the OVX + ligature group (p < 0.01; Figure 5B), whereas the local simvastatin group did not show a significant decrease. Compared to the sham group, the ligature group had a slightly increased serum TRAP5b level (p > 0.05; Figure 5B), and the OVX + ligature group aggravated this trend by 54% (p < 0.01; Figure 5B).

### Biomechanical testing of the femurs

**Figure 6:**
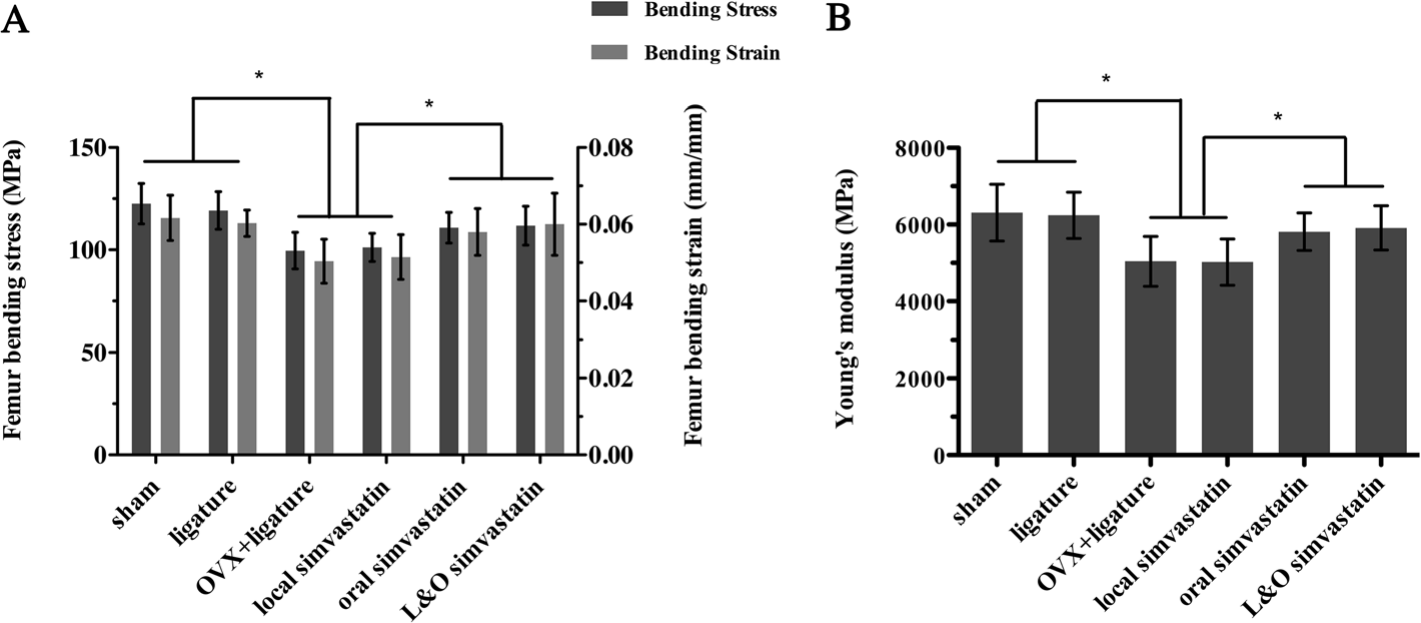
**Effect of simvastatin on biomechanical properties of femurs by the three-point bending test. (A)** Femoral bending stress (MPa) and femoral bending strain (mm/mm). **(B)** Femoral Young’s modulus (MPa). Values are expressed as the mean ± the standard deviation (N = 6; * indicates p < 0.05).

We further checked femur biomechanical properties after simvastatin treatment. After the 2-month treatment, the oral simvastatin and L&O simvastatin groups had significantly greater bending stress (11.7%/11.9%), bending strain (16.1%/16.7%), and Young’s modulus (15.3%/15.9%) compared to the OVX + ligature group (p < 0.05, Figure 6A, B). There was an apparent decrease in bending stress, bending strain, and Young’s modulus in groups having OVX surgery compared to the sham and ligature groups (p < 0.05, Figure 6A, B). However, the local simvastatin group had no preventive effect on the bone’s biomechanical properties (Figure 6A, B).

## Discussion

The worldwide prevalence of periodontitis is between 10% and 15%, and it may reach an astonishing 80% in certain regions [23]. Both periodontitis and osteoporosis are of high prevalence in the elderly. Tezal M et al. [24] found osteoporosis was a risk factor for the loss of periodontal attachment, loss of alveolar crest height, and even loss of tooth. Gomes-Filho IS et al. [25] found that postmenopausal women with osteoporosis were more susceptible to periodontitis. Genco RJ et al. [26] regarded osteoporosis as one of the risk factors for periodontal diseases. Besides, based on our previous animal experiment results (unpublished data), OVX could deteriorate the alveolar bone loss and reduce alveolar crest height of experimental periodontitis in rats. Thus, finding therapeutic agents to alleviate periodontitis, as well as osteoporosis has significant clinical value.

Simvastatin is widely used for cardiovascular diseases. It also has anti-inflammatory, bone-formation promoting, and bone-resorption inhibiting effects. Here, our study further demonstrated the protective effect of simvastatin on an animal model with OVX and experimental periodontitis, suggesting a potential therapeutic benefit of simvastatin for osteoporosis patients with periodontitis. ABL is considered as an indicator of the periodontitis process. In the current study, analysis of the line distance data of CEJ-ABC clearly demonstrated that either local or local combined with oral simvastatin administration had the potential to improve the periodontitis-induced decrease in alveolar crest height. This finding is consistent with previous report, which local simvastatin administration brought a comfortable improvement in treating periodontitis and enhanced bone formation [18,27]. It is noteworthy that oral simvastatin administration failed to prevent the loss of alveolar crest height. This may be due to the systemic metabolism of simvastatin after oral administration. The concentration of simvastatin in the oral administration group may have not reached a high enough level to stimulate alveolar crest bone formation and inhibit inflammation-induced alveolar crest bone resorption, whereas the local administration could easily reach the effective level. Consistent with this finding, histology observation of the region between M1 and M2 in the maxillae showed that the ligature placement caused periodontium destruction, but local simvastatin administration alleviated this process.

In the present study, micro-CT measurement showed that simvastatin treatment moderately increased the BV/TV in OVX rats with periodontitis. Moreover, the structural analysis of Tb.Th and Tb.Sp by micro-CT indicated that local or oral simvastatin administration could only protect against the OVX-induced deterioration of trabecular bone structure to some extent. In 2008, Lee Y et al. [28] demonstrated that simvastatin could effectively augment bone thickness in the mandible. Our findings confirmed this phenomenon and further indicated that local or oral administration of simvastatin could improve the alveolar bone index in the maxilla. When combining local and oral administration, the effect of simvastatin was even more effective and beneficial. All of the above may explain why simvastatin of local or oral administration was able to reduce the periodontitis-associated ABL.

Bone is a metabolically active tissue regulated by osteoblasts and osteoclasts [22,29]. Thus, it was not surprising that the levels of the serum bone resorption marker TRAP5b and the serum bone formation marker OCN increased in OVX rats. Furthermore, our results showed that oral simvastatin administration could increase the level of OCN and reduce the level of TRAP5b. This is in agreement with our findings in the TRAP-stained paraffin sections. Hence, these findings demonstrate that simvastatin might function by stimulating osteoblasts and inhibiting osteoclasts. However, the effects of statins on biochemical markers of bone turnover are still being debated [30]. Different experimental models, animal age, or therapy duration might explain the differences. In addition, it is known that microstructural changes of the bone can affect its biomechanical properties. Bending stress, bending strain, and Young’s modulus are important intrinsic biomechanical properties to describe the bone state [31]. Our data suggested that oral simvastatin administration could enhance systemic bone biomechanical properties, while local administration could not.

Our study confirmed that simvastatin had the potential of recovering alveolar bone loss following experimental periodontitis, and further demonstrated its role in restoring alveolar bone mass induced by OVX. Besides, we also compared the difference between local and oral administration of simvastatin and showed that local simvastatin administration increased alveolar crest height and prevented local alveolar bone loss, but could not change systemic osteoporotic changes, while oral administration improving local or systemic bone osteoporotic changes and bone mass, enhanced bone biomechanical properties, but had no effect on alveolar crest height. Nevertheless, repeated local injections are not suitable for clinical patients; however, a new application method that utilizes methylcellulose gel is promising [32].

## Conclusions

In summary, local and/or oral administration of simvastatin had a positive effect on OVX rats with experimentally induced periodontitis. Whilst the animal study suggests that the combination of local and oral simvastatin administration could be an alternative therapy for periodontitis and osteoporosis, more clinical studies are necessary to explore the effect on these patients with periodontitis and osteoporosis.

## Abbreviations

ABC: Alveolar bone crest
ABL: Alveolar bone loss
BV/TV: Bone volume/tissue volume
CEJ: Cemento-enamel junction
DL: Distolingual
DB: Distobuccal
ELISA: Enzyme-linked immunosorbent assays
H&E: Hematoxylin and eosin
M1: First molar
M2: Second molar
micro-CT: Micro-computed tomography
ML: Mesiolingual
MB: Mesiobuccal
OVX: Ovariectomized
OCN: Osteocalcin
ROI: Region of interest
SD: Standard deviation
TRAP: Tartrate-resistant acid phosphatase
TRAP5b: Tartrate-resistant acid phosphatase 5b
Tb.Th: Trabecular thickness
Tb.Sp: Trabecular separation
Tb.N: Trabecular number
